# Stability and Function of Hippocampal Mossy Fiber Synapses Depend on *Bcl11b/Ctip2*

**DOI:** 10.3389/fnmol.2018.00103

**Published:** 2018-04-05

**Authors:** Elodie De Bruyckere, Ruth Simon, Sigrun Nestel, Bernd Heimrich, Dennis Kätzel, Alexei V. Egorov, Pentao Liu, Nancy A. Jenkins, Neal G. Copeland, Herbert Schwegler, Andreas Draguhn, Stefan Britsch

**Affiliations:** ^1^Institute of Molecular and Cellular Anatomy, Ulm University, Ulm, Germany; ^2^Institute of Anatomy and Cell Biology, Faculty of Medicine, Albert-Ludwigs-University, Freiburg, Germany; ^3^Institute of Applied Physiology, Ulm University, Ulm, Germany; ^4^Institute of Physiology and Pathophysiology, Heidelberg University, Heidelberg, Germany; ^5^School of Biomedical Sciences, Li Ka Shing Faculty of Medicine, University of Hong Kong, Pokfulam, Hong Kong; ^6^Genetics Department, University of Texas, MD Anderson Cancer Center, Houston, TX, United States; ^7^Institute of Anatomy, Otto-von-Guericke-University, Magdeburg, Germany

**Keywords:** *Bcl11b*, transcription factor, hippocampus, mossy fiber boutons, synapses

## Abstract

Structural and functional plasticity of synapses are critical neuronal mechanisms underlying learning and memory. While activity-dependent regulation of synaptic strength has been extensively studied, much less is known about the transcriptional control of synapse maintenance and plasticity. Hippocampal mossy fiber (MF) synapses connect dentate granule cells to CA3 pyramidal neurons and are important for spatial memory formation and consolidation. The transcription factor *Bcl11b/Ctip2* is expressed in dentate granule cells and required for postnatal hippocampal development. Ablation of *Bcl11b/Ctip2* in the adult hippocampus results in impaired adult neurogenesis and spatial memory. The molecular mechanisms underlying the behavioral impairment remained unclear. Here we show that selective deletion of *Bcl11b/Ctip2* in the adult mouse hippocampus leads to a rapid loss of excitatory synapses in CA3 as well as reduced ultrastructural complexity of remaining mossy fiber boutons (MFBs). Moreover, a dramatic decline of long-term potentiation (LTP) of the dentate gyrus-CA3 (DG-CA3) projection is caused by adult loss of *Bcl11b/Ctip2*. Differential transcriptomics revealed the deregulation of genes associated with synaptic transmission in mutants. Together, our data suggest *Bcl11b/Ctip2* to regulate maintenance and function of MF synapses in the adult hippocampus.

## Introduction

The hippocampus plays important roles in consolidation of short- and long-term memory as well as spatial navigation. The hippocampus receives major inputs from the entorhinal cortex to a great extent via the dentate gyrus (DG). Information from sensory and associative cortical regions projects to the hippocampus and is processed in a tri-synaptic loop from the entorhinal cortex via the DG to CA3, CA1 and finally back to the entorhinal cortex. In particular, the DG-CA3 connection (mossy fibers, MFs) has an important function in learning and memory. MFs form one of the most powerful excitatory, glutamatergic “detonator” synapses with CA3 pyramidal cells (Urban et al., 2001; Henze et al., 2002; Mori et al., 2007). Presynaptic mossy fiber boutons (MFBs) exist in three distinct forms: the main large boutons connecting to dendritic spines of the CA3 pyramidal cells in the stratum lucidum and mossy cells of the hilus, smaller *en passant* boutons and filopodial extensions synapsing mainly with GABAergic neurons (Acsády et al., 1998). Together, these synaptic connections provide a powerful excitatory as well as inhibitory feedback circuit. MFBs exhibit variable shapes, sizes, number of mitochondria, synaptic vesicles, active zones and puncta adherentia depending on their developmental stage and functional state (Rollenhagen and Lübke, 2010). They also express specific forms of presynaptic short- and long-term plasticity, involving unique molecular mechanisms (Nicoll and Schmitz, 2005; Südhof, 2017). Transcription factors like members of the CREB family and NF-kB are required for memory formation and synaptic plasticity. The expression of these transcription factors is activated by environmental cues enabling the conversion of short- to long-term memory (Alberini, 2009; Kaltschmidt and Kaltschmidt, 2015; Engelmann and Haenold, 2016).

The zinc finger transcription factor *Bcl11b/Ctip2* is expressed in several brain regions including the hippocampus, neocortex (Arlotta et al., 2005; Chen et al., 2008) and striatum (Arlotta et al., 2008; Desplats et al., 2008). In the hippocampus *Bcl11b/Ctip2* is expressed by granule cells of the DG as well as pyramidal neurons in CA1 and CA2 but not in CA3 regions of the cornu ammonis. Previously, we demonstrated that *Bcl11b/Ctip2* is essential for DG development (Simon et al., 2012). Ablation of *Bcl11b/Ctip2* in the adult hippocampus results in impaired adult neurogenesis and spatial memory (Simon et al., 2016). The underlying molecular mechanisms, however, remained unclear. In the present study we show that selective deletion of *Bcl11b/Ctip2* in the adult mouse hippocampus induces the rapid loss of excitatory synapses in CA3 as well as changes in the ultrastructure of MFBs. Accordingly, weakened MF–CA3 connectivity and a dramatic decline of long-term potentiation (LTP) of the DG-CA3 projection are observed upon adult deletion of *Bcl11b/Ctip2*. Finally, systematic gene expression analyses revealed the enrichment of deregulated genes associated with synaptic transmission in mutants. Together, our data suggest Bcl11b/Ctip2 to be important for the structural maintenance and function of MF synapses in the adult hippocampus.

## Materials and Methods

### Animals

To generate adult induced forebrain-specific *Bcl11b/Ctip2* mutations *Bcl11b*^flox/flox^ mice (Li et al., 2010) were cross-bred with inducible mouse lines, either tetO-Cre and *CaMKIIα*-tTA (Mayford et al., 1996; used only for transcriptome analysis and data presented in Figure 2A, 2 and 4 weeks after induction) or *CaMKIIα-*CreER^T2^ (Erdmann et al., 2007). We confirmed that the two systems were equivalent for the parameters analyzed. Mice of the tet-off system were administered doxycycline (50mg/l; Sigma-Aldrich, cat. #: D9891) in drinking water throughout embryogenesis up to 2 months of age. *Bcl11b*^flox/flox^; *CaMKIIα*-CreER^T2^ (mutant), *Bcl11b*^+/+^; *CaMKIIα*-CreER^T2^ and *Bcl11b*^flox/+^; *CaMKIIα*-CreER^T2^ (controls) animals were injected intraperitoneally 2 mg tamoxifen (stock 10 mg/ml in 1:9 ethanol/peanut oil) at the age of 2 months for five consecutive days. Experiments were performed at 2 and 4 weeks and 2 months after induction of the mutation. Genotyping of the mice was performed by polymerase chain reaction (PCR). All animal experiments were performed according to the German law and were approved by the government offices in Tübingen, Germany.

**Figure 1:**
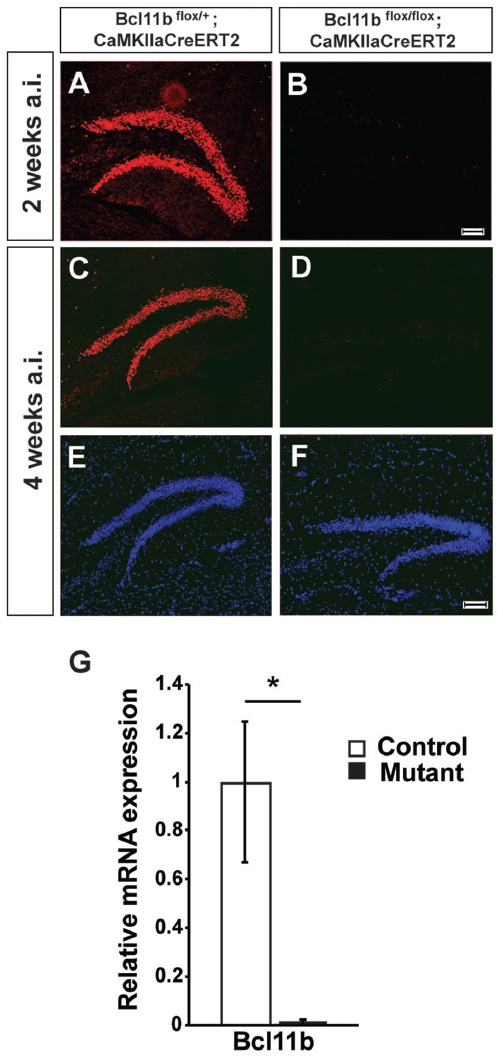
Verification of the tamoxifen-inducible CreER^T2^ system. **(A–F)** Fluorescence staining using a Bcl11b-specific antibody (red) and Dapi (blue) on hippocampal sections of controls **(A,C,E)** and tamoxifen-induced Bcl11b mutants **(B,D,F)** at 2 **(A,B)** and 4 **(C–F)** weeks after induction. **(G)** Quantitative analysis of Bcl11b mRNA expression in the dentate gyrus of controls and tamoxifen-induced Bcl11b mutants at 4 weeks after induction (*n* = 3). Scale bar, 100 μm; error bars, SD; *t*-test, *p < 0.05.

**Figure 2:**
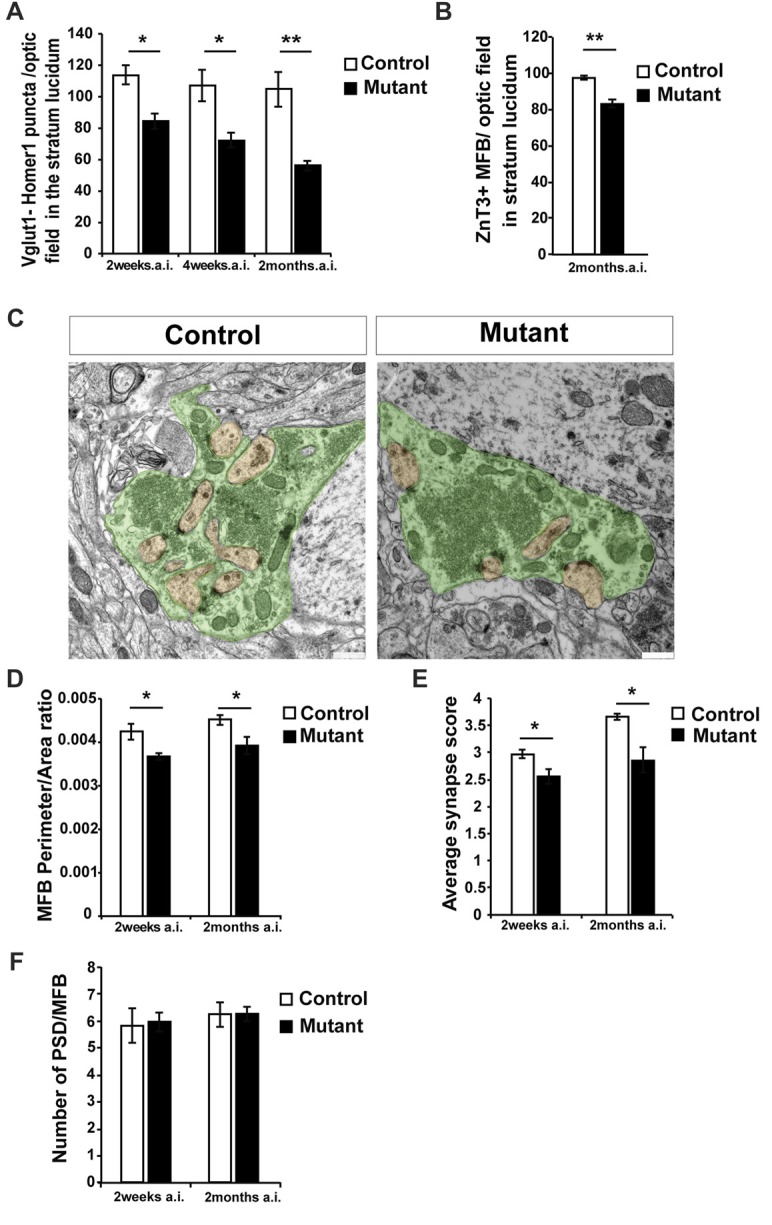
Loss of *Bcl11b/Ctip2* expression impairs mossy fiber (MF) terminals. **(A)** Quantification of overlapping puncta for vesicular glutamate transporter 1 (Vglut1; presynaptic marker of glutamatergic synapses) and Homer1 (postsynaptic marker of glutamatergic synapses) per optic field in the stratum lucidum (*n* = 3; 10 sections per animal). **(B)** Count of Zinc Transporter-3 (ZnT3)-labeled MF terminals per optic field in the stratum lucidum (*n* = 3; 10 sections per animal). **(C)** Representative pictures of control and mutant MFBs; scale bar, 500 nm. **(D)** Perimeter/area ratio of control and mutant MFBs (*n* = 6). **(E)** Average synapse score for mutants and controls (*n* = 4). **(F)** Number of postsynaptic densities per MFB (*n* = 6). Error bars, SEM; Mann-Whitney *U* test, *p < 0.05, **p < 0.01; MFB, mossy fiber bouton; PSD, postsynaptic density; a.i., after induction (of Bcl11b/Ctip2 mutation).

### Immunohistochemistry, Imaging and Analysis

Mouse brains were dissected in ice-cold PBS, cryoprotected in 20% sucrose and embedded in optimal cutting temperature (OCT) compound. Fourteen micrometer coronal cryosections were fixed with 4% paraformaldehyde, permeabilized with 0.2% Triton-X and blocked with 10% calf serum. Mouse anti-Vglut1 (1:100; Synaptic Systems Cat. Nr. 135311), Rabbit anti-Homer1 (1:200; Synaptic Systems Cat. Nr. 160003) and Rabbit anti-ZnT3 (1:200, synaptic systems Cat. Nr. 197003) were incubated for 2 h at 37°C followed by 90 min incubation at 37°C with Cy™3 conjugated AffiniPure donkey anti-mouse IgG (Dianova, Cat. Nr. 715-165-151) and Alexa Fluor^®^488 conjugated AffiniPure donkey anti-rabbit IgG (Dianova, Cat. Nr. 711-165-152; synapse counting) or Cy™3-conjugated AffiniPure donkey anti-rabbit IgG (Dianova, Cat. Nr. 711-165-52; bouton counting).

For Bcl11b/Ctip2 immunostaining, mouse brains were fixed in 4% PFA for 2 h followed by cryoprotection. Fourteen micrometer coronal cryosections were permeabilized with 0.1% Triton-X, blocked with 10% horse serum and then incubated overnight with a guinea pig anti-Bcl11b antibody (1:3000; Simon et al., 2012). Cy™3-conjugated AffiniPure donkey anti-guinea pig IgG (Dianova, Cat. Nr. 706-165-148) was incubated for 2 h at room temperature.

Images were acquired with Leica SP5 laser scanning confocal microscope. Analyses of synapses were performed using ImageJ software.

### Electron Microscopy

Mice were perfused with 1.5% Glutaraldehyde and 4% Paraformaldehyde and postfixed for 4 h. Ultrathin sections (60 nm) were cut (UC 6 from Leica) and stained with lead citrate. Images were acquired using TEM LEO 906 (Zeiss) with the sharp-eye 2k CCD camera and processed with ImageSP (Tröndle, Germany).

Spatial distribution of synaptic vesicles was determined by quantifying the number of vesicles in the vicinity of the active zone according to the following criteria: 0–5 vesicles above active zone = 0; less than 20 vesicles = 1; small group of vesicles (≤200,000 nm^2^) with gap between density and closest vesicle (>100 nm) = 2; small group of vesicles (≤200,000 nm^2^) without gap (≤100 nm) = 3; big group of vesicles (>200,000 nm^2^) with gap (>100 nm) = 4; big group of vesicles (>200,000 nm^2^) without gap (≤100 nm) = 5. Groups with a higher accumulation of vesicles at the active zone were defined by their area. Synapse scoring was based on the description of vesicle distribution and function in MFBs by Rollenhagen and colleagues with the highest score representing the most active synapses (Rollenhagen et al., 2007; Rollenhagen and Lübke, 2010).

### Electrophysiology

#### Input/Output and Short-Term Plasticity

Animals were deeply anesthetized with CO_2_ and decapitated 2 months after induction at the age of 4 months. Brains were transferred in ice-cold artificial cerebrospinal fluid (ACSF) containing 124 mM NaCl, 3 mM KCl, 1.8 mM MgSO_4_, 1.6 mM CaCl_2_, 10 mM glucose, 1.25 mM NaH_2_PO_4_, 26 mM NaHCO_3_ and saturated with carbogen (95% O_2_/5% CO_2_, pH 7.4 at 34°C). 450 μm horizontal hippocampal slices were cut in ice-cold ACSF using a vibratome (Leica VT1200S) and transferred into an interface type chamber, also known as “Haas”-type chamber (Haas et al., 1979), perfused with ACSF at a rate of 1.52 ml/min, and maintained at 34 ± 1°C. Slices were left to recover for a minimum of 2 h prior to the recordings. Extracellular field potentials (fp) were amplified 100× with an EXT 10-2F amplifier (npi electronics, Tamm, Germany). Signals were low-pass filtered at 2 kHz and high-pass filtered at 0.3 Hz, digitized at 20 kHz with an analog-to-digital converter (ADC; model MICRO 1401 mkII, Cambridge Electronic Design (CED), Cambridge, UK) and saved on a computer using Spike2 software (CED, Cambridge, UK) for offline analysis. Recordings were carried out by placing a glass micropipette (tip diameter 3–5 μm) filled with ACSF in the stratum lucidum of the CA3. To stimulate MF-field excitatory post-synaptic potentials (fEPSP), a bipolar electrode was placed within the inner border of the granule cell layer. Pulses of 0.1 ms were delivered with an Iso-Flex stimulus isolator (AMPI, Jerusalem, Israel) at intervals of 20 s. Input/output analysis was performed by measuring the fEPSP amplitude in response to a range of stimulations in Volt. To assess short-term plasticity, the stimulation was set for fEPSP = 60% of max-fEPSP for each slice, and paired-pulse stimulations were applied with a range of time intervals. Facilitation (or depression) for each time interval was calculated as percentage of the second fEPSP amplitude divided by the amplitude of the first fEPSP.

#### Long-Term Plasticity

Animals at 2 months after induction (4 months old) were deeply anesthetized with isofluorane and decapitated. 350 μm horizontal brain slices of the left hemisphere were prepared in ice-cold modified ACSF solution (pH 7.4) containing 135 mM N-methyl-D-glucamine (NMDG), 10 mM D-glucose, 1.5 mM MgCl_2_, 0.5 mM CaCl_2_, 1 mM KCl, 1.2 mM KH_2_PO_4_, 20 mM choline bicarbonate and saturated with carbogen gas (95% O_2_/5% CO_2_). The cutting angle was chosen to improve the preservation of MFs, as previously described (Bischofberger et al., 2006). Slices were incubated for 30–45 min at 36°C and subsequently at room temperature in ACSF containing: 125 mM NaCl, 2.5 mM KCl, 25 mM NaHCO_3_, 1.25 mM NaH_2_PO_4_, 25 mM glucose, 2 mM MgCl_2_, 2 mM CaCl_2_ and saturated with carbogen. Not less than 1 h after the preparation of the slices, single slices were transferred into the recording chamber, where they were perfused continuously with ACSF. Recordings were performed at room temperature. The stimulation current for all subsequent experiments in a given slice was set to obtain a baseline fEPSP with an amplitude of 0.2 mV (Ben-Simon et al., 2015), and only slices exhibiting paired-pulse facilitation of the fEPSP (25 Hz) were used for further recordings. Short-term plasticity during five consecutive stimulations at 25 Hz (burst) was also recorded from these slices. LTP was induced by three trains of 100 stimulation pulses at 100 Hz (high frequency stimulation, HFS), repeated every 8 s. Changes in the fEPSP amplitude were calculated in percentage of the average baseline fEPSP ((average amplitude of the fEPSP before HFS − average amplitude of the fEPSP in a given interval after HFS)/(average amplitude of the fEPSP before HFS)). Bath application of 1 μM of the mGlu2/3 agonist DCG-IV (Tocris, Bioscience) was performed after LTP experiments, and only the recordings with putative MF-EPSP reduced by at least 60% were used for LTP analysis (Yoshino et al., 1996).

### Microarray, qRT-PCR and Chromatin Immunoprecipitation (ChIP) Analysis

The granule cell layer of the DG was isolated by laser capture microdissection (Zeiss Axio Observer 2.1 with RoboMover and PALM microbeam; PALM software 4.6) 4 weeks after induction of the mutation. RNA isolation, microarray analysis and quantitative real-time PCR were described in Simon et al. (2012). Briefly, RNA of control and mutant DG (*n* = 3) was isolated and integrity and purity was determined by an Agilent Bioanalyzer and a Nano-Drop spectrophotometer. Transcriptome analysis was performed using BRBArrayToolsdeveloped by Dr. Richard Simon and BRB-ArrayTools Development Team^1^. The data obtained in our microarray experiment were deposited at the GEO website under accession number GEO: GSE111494. Microarray data were further analyzed by an overrepresentation test performed with PANTHER version 10.0 (Mi et al., 2013, 2016). For RT-PCR the following primers were used: *Bcl11b*: 5′-TGGATGCCAGTGTGAGTTGT-3′ and 5′-AATTCATGAGTGGGGACTGC-3′, *C1ql2*: 5′- TTGGCAATCACTACGACCCC -3′ and 5′-CCCGAGAACGTGCTGTACTT-3′, *Kif17*: 5′-GGCTGAGAAGCAGTTGATCC-3 and 5′-GGCCTCTGTCTCCTTCTCCT-3, *Pdyn*: 5′-ACTGTCCCGCCTCAAAACTA-3′ and 5′-TGGAGATGGGGAGGTGAAAC-3′, *Penk*: 5′-CCCAGGCGACATCAATTTCC-3′ and 5′-GGCTCTCATCCTGTTTGCTG-3′, *Ptgs2*: 5′-AAGCGAGGACCTGGGTTCAC-3′ and 5′-ACACCTCTCCACCAATGACCTG-3′, *Sema5B*: 5′-CTGGTCACTGTGGTCTGAGT-3′ and 5′-CACACTGGAAGCAGGTAGGA-3′, *Tanc1*: 5′-ACTCCCAACGTGAAGGTGAG-3 and 5′-CCCCTTCTTGATGAGCAGAC-3 as well as *Gapdh* as internal control: 5′-CCAGAGCTGAACGGGAAG-3′ and 5′-TGCTGTTGAAGTCGCAGG-3′. The relative mRNA expression of *Gapdh* was quantified and used for normalization. Chromatin immunoprecipitation (ChIP) assays are described elsewhere (Simon et al., 2012). Briefly, ChIP assays were performed on hippocampal tissue of two adult wild-type animals using a specific antibody recognizing Bcl11b (Abcam, Cat. # ab18465) as well as an anti-RNA polymerase II antibody (Abcam, Cat. #ab817) as positive control and IgG from mouse serum (Sigma, Cat. # 15381) as negative control. The precipitated DNA was analyzed by quantitative PCR assays using the following primers: C1ql2: 5′-GGTGGTCTGCGAGAGGAG-3′ and 5′-CTGTCATCAGCTCCCATCC-3′; Sema5b: 5′-ACTAGCAACCGGTCAGCTCT-3′ and 5′-TCTCTGCCATCCCCATCTC-3′; Gapdh: 5′-AAGGCTGGTGCTGTGGAGAAACTG-3′ and 5′-GTCCCCTTGCAACATACATAACTG-3′. ChIP and qRT-PCR data were analyzed with the 2^−ΔΔCT^ method (Livak and Schmittgen, 2001).

### Statistical Analysis

Results are expressed as the mean ± SEM or ± SD (quantitative RT-PCR). Comparisons between groups were made by an unpaired two-tailed Student’s *t*-test or analyzed using the 2^−ΔΔCT^ method (Livak and Schmittgen, 2001). Normality was tested prior to application of parametric tests. Synapse score data were analyzed by Mann-Whitney *U*-tests and data for the 25 Hz burst experiment were analyzed by analysis of variance (ANOVA).

## Results

### Induction of the *Bcl11b/Ctip2* Mutation in Adult Animals

To analyze *Bcl11b/Ctip2* regulation of hippocampal synapse plasticity we employed two inducible systems under control of the forebrain specific *CaMKIIa*-promoter, the doxycycline dependent tet-off and the tamoxifen-inducible CreER^T2^ systems (Mayford et al., 1996; Erdmann et al., 2007). Forebrain specific ablation of Bcl11b using the tet-off system was confirmed previously (Simon et al., 2016). To assess the efficiency of the CreER^T2^ system animals were injected with tamoxifen at the age of 2 months and analyzed 4 weeks after the induction of the *Bcl11b/Ctip2* mutation corresponding with the time point of the microarray analysis. *Bcl11b/Ctip2* expression was not detected in the *Bcl11b*^flox/flox^; *CaMKIIa*CreER^T2^ mutant either by immunohistochemistry (Figures 1C–F) or by qRT-PCR (control: 1 ± 0.248/0.331 SD; mutant: 0.015 ± 0.008/0.015 SD; *p* < 0.05; *n* = 3; Figure 1G) at 4 weeks after induction of the mutation. Experiments were also performed as early as 2 weeks after induction of the Bcl11b/Ctip2 mutation therefore we confirmed the depletion of Bcl11b/Ctip2 protein at this earlier time point (Figures 1A,B). Thus, both strategies are equally efficient to induce Bcl11b/Ctip2 ablation specifically in the adult hippocampus.

### *Bcl11b/Ctip2* Expression Is Required for Synapse Maintenance

In order to quantify glutamatergic synapses within CA3, sections were labeled for the presynaptic marker vesicular glutamate transporter 1 (Vglut1) and the postsynaptic protein Homer1 (Supplementary Figures S1A,B). Co-localization of these markers was considered to represent glutamatergic synapses (Grabrucker et al., 2014; Nikitczuk et al., 2014). Counting puncta co-expressing Vglut1 and Homer1 in stratum lucidum of CA3 revealed a strong decrease in the density of glutamatergic synapses in *Bcl11b/Ctip2* mutant mice by 25.9% as early as 2 weeks after induction (wai; control: 113.86 ± 6.15 SEM; mutant: 84.37 ± 4.94 SEM; *p* < 0.05; *n* = 3) progressing further to 32.7% (control: 107.21 ± 10.21 SEM; mutant: 72.11 ± 4.75 SEM; *p* < 0.05; *n* = 3) and 48.1% (control: 107.71 ± 11.16 SEM; mutant: 55.9% ± 2.93 SEM; *p* < 0.05; *n* = 3; 10 sections per animal) at 4 weeks and 2 months after induction (mai), respectively (Figure 2A). Further analysis at 2 months after induction revealed a reduced number of the MF terminals stained for the Zinc Transporter-3 (ZnT3; control: 97.68 ± 1.15 SEM; mutant: 83.28 ± 2.29 SEM; *p* = 0.005; *n* = 3; 10 sections per animal; Figure 2B; Supplementary Figures S1C,D), a molecule required for Zn^2+^ accumulation in presynaptic vesicles of glutamatergic neurons and enriched in MF terminals (Wenzel et al., 1997).

To determine ultrastructural changes, we examined control and adult-induced *Bcl11b/Ctip2* mutant MFBs by electron microscopy. In adult-induced *Bcl11b/Ctip2* mutants, MFBs exhibited a change in shape from a highly folded to a more even rounded structure with fewer invaginated spines (Figure 2C) resulting in a reduction of the perimeter/area ratio in the *Bcl11b/Ctip2* mutant (2 wai: control: 0.00424 ± 0.00018 SEM; mutant: 0.00367 ± 0.00008 SEM; *p* < 0.05; *n* = 6; 2 mai: control: 0.00451 ± 0.00012 SEM; mutant: 0.00392 ± 0.0002 SEM; *p* < 0.05; *n* = 6; Figure 2D). Surprisingly, no difference in the number of post-synaptic densities per MFB was observed (2 wai: control: 5.839 ± 0.651 SEM; mutant: 5.969 ± 0.362 SEM; *n* = 6; *p* = 0.86; 2 mai: control: 6.245 ± 0.465 SEM; mutant: 6.265 ± 0.282 SEM; *n* = 6; *p* = 0.97; Figure 2F).

Accumulation of synaptic vesicles in the vicinity of active zones represents one important structural key component for synapse activity. Depending on their location relative to the active zone the synaptic vesicles are divided into a readily releasable, a recycling and a reserve pool (Rollenhagen et al., 2007; Rollenhagen and Lübke, 2010). As a proxy for the readily releasable pool of vesicles, we determined the number and spatial distribution of vesicles in the vicinity of MF synapses at 2 weeks and 2 months after induction of the *Bcl11b/Ctip2* mutation. While the overall number of synaptic vesicles per MFB is not changed (data not shown) we found a redistribution of synaptic vesicles. Due to fewer synaptic vesicles in the vicinity of the active zone, adult-induced *Bcl11b/Ctip2* mutants exhibited a lower synapse score suggesting reduced synapse activity (2 wai: control: 2.975 ± 0.081 SEM; mutant: 2.553 ± 0.131 SEM; *p* < 0.05; *n* = 3; 2 mai: control: 3.653 ± 0.063 SEM; mutant: 2.86 ± 0.23 SEM; *p* < 0.05; *n* = 3; Figure 2E).

### Ablation of *Bcl11b/Ctip2* Prevents Long-Term Potentiation

**Figure 3:**
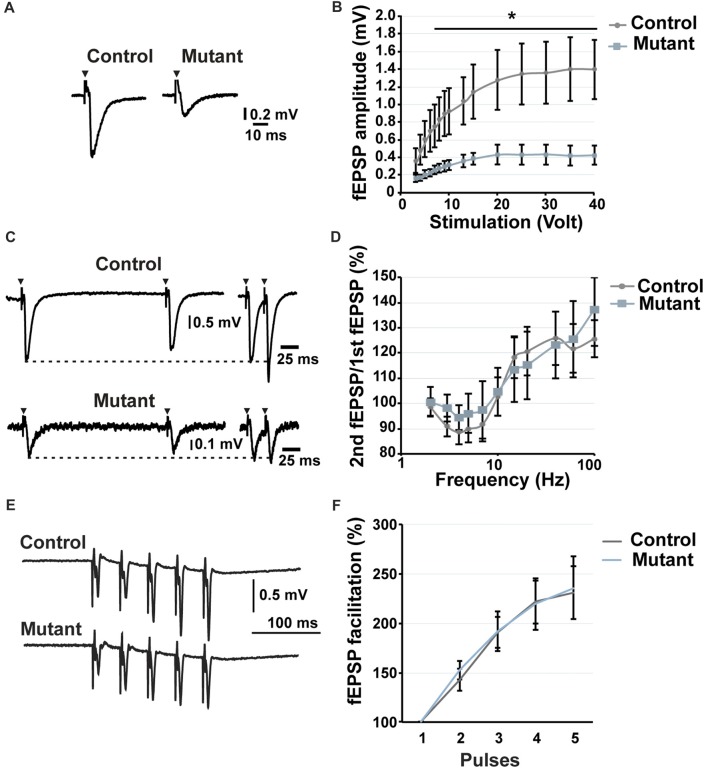
Analysis of short-term potentiation at 2 months after induction. **(A)** Representative traces of fEPSP recorded in the stratum lucidum in response to a stimulation of 20 volt. **(B)** Input/output curve showing the fEPSP amplitude depending on stimulation (*n* = 9). **(C)** Representative traces of fEPSP in the stratum lucidum induced by electrical stimulation of MFs at 5 Hz (left) and 40 Hz (right). **(D)** Paired-pulse analysis demonstrating the facilitation or the inhibition of the second fEPSP compared to the first one (*n* = 9). **(E)** Representative traces of fEPSP in response to five consecutive stimulations at 25 Hz. **(F)** Facilitation of the fEPSP in response to five consecutive stimulations at 25 Hz. *n* = 5, control; *n* = 7, mutant; error bars, SEM; *t*-test, *p < 0.05; fEPSP, field excitatory postsynaptic potential.

**Figure 4:**
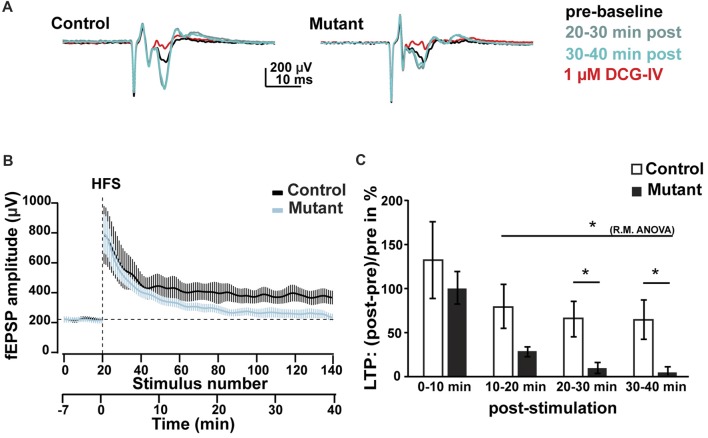
Analysis of LTP at 2 months after induction. **(A)** Representative average traces of baselines before LTP induction by HFS (pre-baseline, in black), 20–30 min after LTP induction (20–30 min post, in light blue), 30–40 min after LTP induction (30–40 min post, in green) and after DCG-IV application (1 μM DCG-IV, in red). **(B)** Time course of the fEPSP amplitude during LTP experiment. **(C)** Analysis of the facilitation of fEPSP at 0–10, 10–20, 20–30 and 30–40 min after HFS. *n* = 5, control; *n* = 7, mutant; error bars, SEM; *t*-test, *p < 0.05; fEPSP, field excitatory postsynaptic potential; HFS, high frequency stimulation; LTP, long-term potentiation; R.M.ANOVA, repeated measure ANOVA.

To determine whether loss of Bcl11b/Ctip2 impairs MF synapse function, we performed field-recordings at 2 mai in the stratum lucidum while MFs were stimulated by a bipolar field electrode placed close to the granule cell layer. Initial measurements determined that the rate of MF neurotransmission was not affected (Supplementary Figure S2). Input-output analysis demonstrated a weakened DG-CA3 connectivity in *Bcl11b/Ctip2* mutants as characterized by lower fEPSP amplitudes in response to MF stimulation (Figures 3A,B). Short-term plasticity of MF synapses was not affected by the loss of *Bcl11b/Ctip2* expression, as the paired-pulse analysis did not reveal any difference between controls and mutants (Figures 3C,D). In addition, no defect of facilitation was observed when applying 5 consecutive stimulations at a frequency of 25 Hz (Figures 3E,F). However, *Bcl11b/Ctip2* mutant mice presented a dramatic loss of LTP compared to controls as measured by the relative increase of the fEPSP at two different time intervals after HFS (0–10 min: control: 131.9% ± 43.8% SEM; mutant: 100.3% ± 18.2% SEM; *p* = 0.5; 10–20 min: control: 79.4% ± 25.5% SEM; mutant: 28.2% ± 5.7% SEM; *p* = 0.11; 20–30 min: control: 65.2% ± 20.0% SEM; mutant: 9.6% ± 6.1% SEM; *p* < 0.05; 30–40 min: 64.2% ± 21.9% SEM; mutant: 5.2% ± 15.2% SEM; *p* < 0.05; control: *n* = 5; mutant: *n* = 7; Figures 4A–C). Furthermore, performing a pair-test comparison among the different time points after HFS revealed a significant effect of the genotype on LTP between time points 10–20 and 30–40 (*p* < 0.05; Figure 4C).

### Transcriptome Analysis Reveals an Overrepresentation of Synapse Transmission Genes

**Figure 5:**
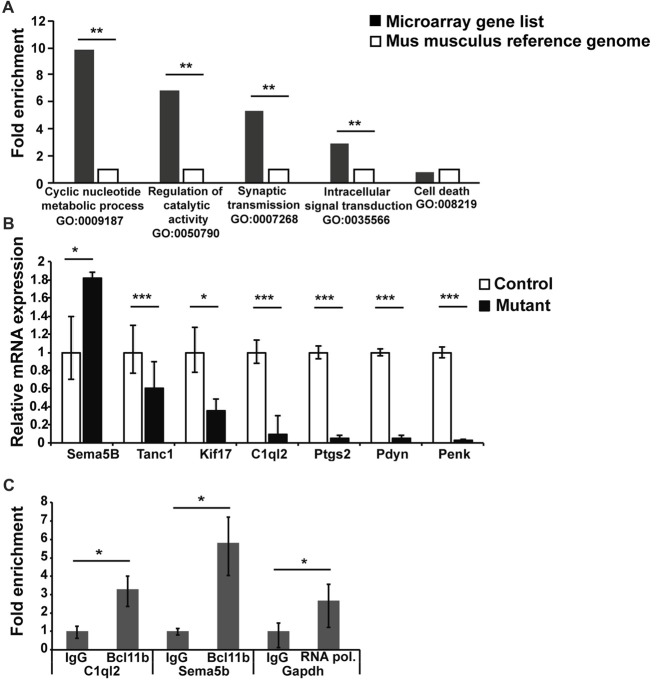
Transcriptome analysis of DG granule cells of adult-induced *Bcl11b/Ctip2* mutants. **(A)** Gene ontology analysis reveals overrepresentation of “Cyclic nucleotide metabolite process GO:0009187; Regulation of catalytic activity GO:0050790; Synaptic transmission GO:0007268; Intracellular signal transduction GO:0035566” but not “Cell death GO:008219” as determined by PANTHER version 10.0. **(B)** Verification of potential *Bcl11b/Ctip2* target genes by qRT-polymerase chain reaction (PCR) at 4 weeks after induction of the mutation (*n* = 4). **(C)** Representative data of 3 independent chromatin immunoprecipitation (ChIP) assays determining the direct interaction of *Bcl11b/Ctip2* with C1ql2 and Sema5B promoter regions, respectively. ChIP assays were performed on hippocampal tissue of 3 months old wild-type animals employing a *Bcl11b/Ctip2*-specific antibody, IgG as a negative control and an RNA polymerase II (RNA pol)-specific antibody as positive control. Direct interaction was determined by qPCR using specific primers for the C1ql2, Sema5b and Gapdh promoters. Error bars, SD; *p < 0.05; **p < 0.01; ***p < 0.001.

To explore the mechanism(s) of *Bcl11b/Ctip2* regulation of MF synapse maintenance and function, we carried out a transcriptome analysis on dentate granule cells 4 weeks after the induction of the *Bcl11b/Ctip2* mutation identifying 264 deregulated genes. These genes were subsequently compared to a Mus musculus reference genome (GRCm38) using an overrepresentation test by PANTHER version 10.0 (Mi et al., 2013, 2016). The analysis revealed a substantial overrepresentation of the gene ontology annotation “Cyclic nucleotide metabolite process GO:0009187 (*p* = 0.009); Regulation of catalytic activity GO:0050790 (*p* = 0.006); Synaptic transmission GO:0007268 (*p* = 0.006); Intracellular signal transduction GO:0035566 (*p* = 0.008)” but not as an example of “Cell death GO:008219 (*p* = 1)” (Figure 5A) further supporting the role of *Bcl11b/Ctip2* in the regulation of synapse function. Several genes of interest involved in axon guidance, synapse formation and maintenance as well as regulation of synapse transmission were further verified by quantitative RT-PCR at 4 weeks after the induction of the mutation. We confirmed the upregulation of mRNA expression of the axon guidance molecule Sema5B (control: 1 ± 0.4/0.3; mutant: 1.82 ± 0.06/0.06; *p* < 0.05) in the absence of Bcl11b/Ctip2, and the loss of mRNA expression of Tanc1 (control: 1 ± 0.03/0.023; mutant: 0.6 ± 0.3/0.21; *p* < 0.001), Kif17 (control: 1 ± 0.28/0.22; mutant: 0.36 ± 0.13/0.1; *p* < 0.05), C1ql2 (control: 1 ± 0.14/0.12; mutant: 0.1 ± 0.2/0.07; *p* < 0.00), Ptgs2 (control: 1 ± 0.07/0.075; mutant: 0.056 ± 0025/0.05; *p* < 0.001), Pdyn (prodynorphin; control: 1 ± 0.04/0.04; mutant: 0.05 ± 0.03/0.03; *p* < 0.001) as well as Penk (proenkephalin; control: 1 ± 0.06/0.06; mutant: 0.032 ± 0.005/0.006; *p* < 0.001; Figure 5B).

Besides Sema5B we considered C1ql2, Ptgs2, Pdyn and Penk the most promising Bcl11b/Ctip2 downstream candidate genes. It was shown that Ptgs2 inhibits Vglut expression and Pdyn and Penk are involved in the inhibition of synapse transmission (Jaffe and Gutiérrez, 2007; Llorente et al., 2013). Because all three genes are downregulated in the adult-induced Bcl11b/Ctip2 mutant we concluded that they are unlikely directly involved in the Bcl11b/Ctip2 synapse phenotype. Sema5B and C1ql2 on the other hand play critical roles in the regulation of synapse stability and function (O’Connor et al., 2009; Yuzaki, 2010, 2017). Furthermore, the expression of Sema5B and C1ql2 is restricted to dentate granule cells within the hippocampus. Thus, we focused on these two genes for further analysis. To determine whether Bcl11b/Ctip2 directly regulates the expression of Sema5B and C1ql2, we performed ChIP assays on 3 months old wild-type adult hippocampal tissue using a Bcl11b/Ctip2 specific antibody followed by qPCR. Genomic analysis by Ensemble.org of the Sema5B and C1ql2 loci revealed potential promoter sites at −81887 to −81638 and −751 to −604, relative to the respective transcription start site. The C1ql2 promoter region contains two copies of the putative Bcl11b/Ctip2 binding site TGGGC (Cismasiu et al., 2006) which are not present in the Sema5B promoter. qPCR data employing specific primers revealed for C1ql2 a 3.29 (IgG: 1 ± 0.28/0.38; Bcl11b 3.29 ± 0.72/0.93; *p* < 0.05) and for Sema5B a 5.83 (IgG: 1 ± 0.17/0.205; Bcl11b 5.83 ± 1.37/1.79; *p* < 0.05) fold enrichment of Bcl11b/Ctip2 precipitated promoter DNA in comparison to IgG only (Figure 5C) suggesting a direct binding of Bcl11b/Ctip2 to these promoter regions.

## Discussion

Here we report that selective ablation of the transcription factor *Bcl11b/Ctip2* in the adult forebrain leads to impaired DG-CA3 connectivity as demonstrated by a reduction of synapses, reshaping of MFBs and loss of LTP.

Adaptive behavior depends on synaptic plasticity allowing to process information in an experience-dependent way. Changes in synapse plasticity have been reported in a number of neurological disorders like Alzheimer’s, epilepsy and mental retardation (Patrylo et al., 1999; Lanore et al., 2012; Wilke et al., 2014; Martin et al., 2017; Scharkowski et al., 2018). Loss of Bcl11b/Ctip2 in adulthood leads to a reduction of synapses in stratum lucidum of CA3. The analysis of ultrastructural changes of MFBs of the adult-induced *Bcl11b/Ctip2* mutant revealed a significant change of the area/perimeter ratio as well as an overall reshaping of the boutons (Figures 2C,D). Surprisingly, no change in the number of synapses and postsynaptic density (PSD) was observed in single boutons suggesting that the overall reduction of synapses might be due to either a reduced expression of the markers used, Vglut1 and Homer1, or a decrease in the number of MFBs. The latter is supported by a reduced number of ZnT3 labeled MF terminals in *Bcl11b/Ctip2* mutants (Figure 2B). Furthermore, we observed a change in synaptic vesicle distribution in the mutant MFBs of our mouse model which might be due to reduced activity or altered spatial organization of the presynaptic terminals. Ablation of Bcl11b/Ctip2 in adulthood causes a reduction in the number of thorny excrescences, the terminals for MFs in the CA3 where no *Bcl11b/Ctip2* expression occurs (Simon et al., 2016). The morphology and plasticity of thorny excrescences can be altered by a variety of factors. MFBs and thorny excrescences are influencing each other bi-directionally depending on their activity involving, among other factors, presynaptic synaptoporin expression (Lee et al., 2013). Therefore, it is possible that the observed reduction of MF synapses causes the previously observed reduction of thorny excrescences.

We demonstrated that loss of *Bcl11b/Ctip2* expression in adulthood leads to an increase of apoptosis and decrease of differentiation at 2 and 4 months after induction causing a reduced size and cell number of the DG (Simon et al., 2016). No significant change of these parameters was observed at 2 weeks after induction between control and mutant animals (unpublished data). This is in contrast to the observed reduction in the number of synapses occurring already at 2 weeks after the induction of the *Bcl11b/Ctip2* mutation preceding the onset of cell death. Synapse reduction in turn causes a malfunction of neurons leading in due course to acute cell death or increased delayed apoptotic cell loss (Brady and Morfini, 2010), and conversely, synaptic activity can prevent apoptosis by regulating the caspase pathway or members of the Bcl-2 family (Léveillé et al., 2010; Jonas, 2014). Thus, in our mouse model it appears that apoptosis is not the cause but the consequence of impaired synaptic function and integrity. Our data suggest that loss of synapses is the first indicator of changes in DG-CA3 connectivity after ablation of Bcl11b/Ctip2, indicating an important function of this factor in synapse formation and maintenance.

Reduction of the number of synapses causes reduced signal transmission and processing. Adult-induced *Bcl11b/Ctip2* mutants exhibit a loss of LTP but no change in short-term potentiation. Hippocampal LTP is essential for learning and memory (Hagena and Manahan-Vaughan, 2011). Therefore, the observed loss of LTP is most likely the cause of the observed impairment in learning and memory formation in adult-induced *Bcl11b/Ctip2* mutants (Simon et al., 2016). LTP of MFs does not depend on NMDA receptors but is carried out by presynaptic mechanisms like the activation of presynaptic kainate receptors (Contractor et al., 2001; Lauri et al., 2001; Bortolotto et al., 2003). Presynaptic kainate receptors are also involved in short-term potentiation, which is unchanged in our mouse model suggesting that Bcl11b/Ctip2 most likely regulates LTP by different mechanisms. This may include altered regulation of pre-synaptic calcium dynamics which plays an important role in MF LTP (Castillo et al., 1994) and/or the impairment of transcriptional regulation of synaptic plasticity (Alberini, 2009; Kaltschmidt and Kaltschmidt, 2015; Engelmann and Haenold, 2016). Contrary to short-term memory which provides information for a brief time without neuronal changes, long-term memory is expressed by changes in neuronal pathways that allow storage of information (Costa-Mattioli et al., 2009). These changes require *de novo* gene expression in a time dependent manner. The link between synaptic activity and nuclear activity, that allows the conversion from short- to long-term plasticity and memory, involves a number of transcription factors as, for example, CREB and NF-kB. These transcription factors are activated by environmental cues and in turn activate their target genes to facilitate memory storage (Kaltschmidt and Kaltschmidt, 2015). Failing to activate *de novo* gene expression can cause neurological disorders as was shown for the Foxp1 transcription factor. Loss of Foxp1, specifically in pyramidal cells of the neocortex and CA1/2, is sufficient to cause autism spectrum disorder and intellectual disability behavior by impairing hippocampal LTP maintenance (Araujo et al., 2017). A number of neurodegenerative and neurodevelopmental diseases, e.g., Alzheimer’s and autism spectrum disorder, are associated with a decrease in synaptic density suggesting that loss of Bcl11b/Ctip2 could contribute to these neurological disorders. Recently, the first patient with *Bcl11b/Ctip2* mutations was examined revealing an overall developmental delay with speech impairment and intellectual disabilities (Punwani et al., 2016). It remains to be determined which molecular mechanism(s) regulated by *Bcl11b/Ctip2* contribute to these disorders.

The downstream functions of Bcl11b/Ctip2 at the MF synapse are not yet clear. Loss of Bcl11b/Ctip2 leads to both, structural and functional impairments of MF synapses. This raises the question whether dysfunctional synapses are responsible for the ultrastructural changes observed in the *Bcl11b/Ctip2* mutant MFB and/or vice versa. Ultrastructural changes like the reduction of the pool of readily releasable vesicles at the presynaptic terminals, can alter physiological properties of MF synapses (Midorikawa and Sakaba, 2017). On the other hand, it has been well established, especially during development, that synaptic activity affects the structure of synaptic elements and that defective or reduced activity can lead to synapse elimination (Mezey et al., 2004; Piatti et al., 2011; Römer et al., 2011). We have shown that Bcl11b/Ctip2 is required for postnatal development of the DG. Furthermore, we demonstrated that desmoplakin, a direct downstream target gene of Bcl11b/Ctip2 is essential for postnatal neurogenesis but not for learning and memory (Simon et al., 2012). To determine the mechanism(s) by which *Bcl11b/Ctip2* regulates synapse formation and maintenance, we performed a transcriptome analysis revealing the deregulation of genes involved in synaptic transmission and signaling. Our data demonstrate the direct regulation of Sema5B and C1ql2 expression by Bcl11b/Ctip2 (Figure 5C). Previously it was shown that Sema5B regulates the elimination of hippocampal synaptic connections in cultured neurons (O’Connor et al., 2009). In our adult-induced mouse model Sema5B expression is upregulated in the *Bcl11b/Ctip2* mutant coinciding with the loss of synapses. Another member of the semaphorin family, Sema3F, is involved in pruning of MF axons during postnatal development of the DG (Bagri et al., 2003). We have shown that depletion of Bcl11b/Ctip2 during postnatal development leads to impaired pruning (Simon et al., 2012). It is possible that Bcl11b/Ctip2 regulates similar mechanisms to eliminate superfluous and/or not functional synapses during postnatal DG development and adulthood by interacting with different members of the Semaphorin family. Recently, members of the C1q complement family were shown to be involved in synapse organization, modulation and maintenance (Yuzaki, 2017). These molecules are highly expressed in the brain, in particular C1ql2 and 3 in DG granule cells. C1qls are presynaptically released into the synaptic cleft between MFs and CA3 pyramidal neurons to determine transsynaptically the position of the postsynaptic kainate receptors (Matsuda et al., 2016; Matsuda, 2017). Among other genes C1ql2 expression is down-regulated in *Bcl11b/Ctip2* adult-induced mutants, and Bcl11b/Ctip2 protein physically binds to C1ql2 promotor sequences strongly suggesting that Bcl11b/Ctip2 acts on MF synapses through transcriptional regulation of C1ql2. Future analysis of Bcl11b/Ctip2 interaction with C1ql2 as well as other potential target genes will contribute to determine the mechanism(s) of Bcl11b/Ctip2 regulation in synapse plasticity.

## Author Contributions

EB, DK, BH, RS, HS, AD and SB designed the experiments. EB, RS, SN, BH, DK and AVE conducted the experiments and analyzed the data. PL, NAJ and NGC provided *Bcl11bflox* mice. RS, EB and SB wrote the manuscript.

## Conflict of Interest Statement

The authors declare that the research was conducted in the absence of any commercial or financial relationships that could be construed as a potential conflict of interest.

## Footnotes

^1^http://linus.nci.nih.gov/BRB-ArrayTools.html

## Supplementary Material

FIGURE S1 Immunofluorescence staining of MF terminals at 2 months after the induction of the *Bcl11b/Ctip2* mutation. **(A,B)** Mossy fiber synapse staining employing Vglut1 (red) and Homer1 (green) specific antibodies. Puncta representing Vglut1 and Homer1 co-localization were counted. **(C,D)** Mossy fiber bouton staining using ZnT3- specific antibody (red). Dapi was used for nuclear counter staining. Inlets representing an enlargement of areas of interest. Confocal images taken at 40×, zoom 2 magnification. Scale bar, 10μm.

FIGURE S2 Time related analysis of mossy fiber neurotransmission. Measurement of the time separating stimulation of the mossy fiber to fEPSP maximal amplitude.
