# Supplementary figures and images for: Stability and Function of Hippocampal Mossy Fiber Synapses Depend on Bcl11b/Ctip2
Source: Front Mol Neurosci. 2018 Apr 5;11:103. doi: 10.3389/fnmol.2018.00103 (PMC5895709; doi:10.3389/fnmol.2018.00103)

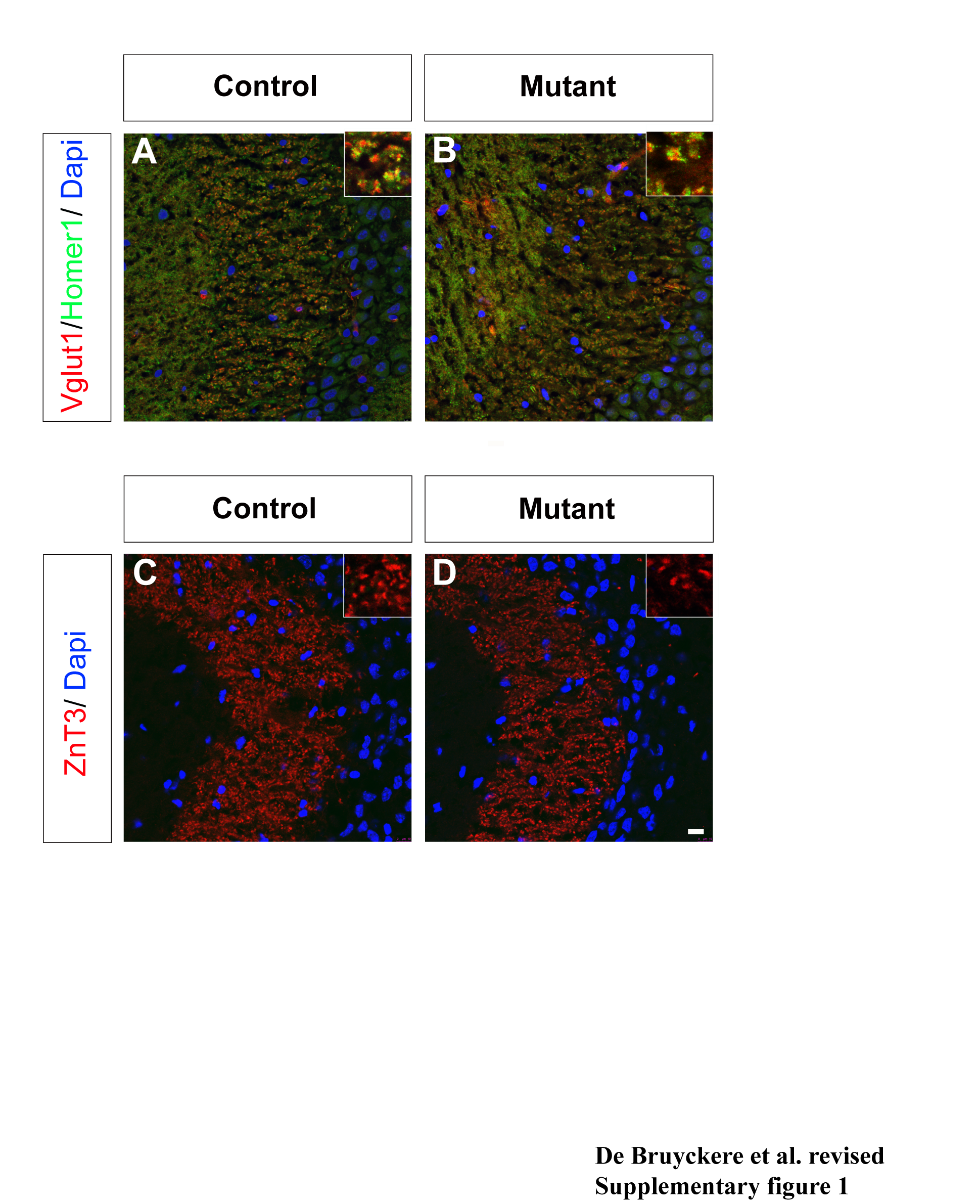

Supplement: FIGURE S1 — Immunofluorescence staining of MF terminals at 2 months after the induction of the Bcl11b/Ctip2 mutation. (A,B) Mossy fiber synapse staining employing Vglut1 (red) and Homer1 (green) specific antibodies. Puncta representing Vglut1 and Homer1 co-localization were counted. (C,D) Mossy fiber bouton staining using ZnT3- specific antibody (red). Dapi was used for nuclear counter staining. Inlets representing an enlargement of areas of interest. Confocal images taken at 40×, zoom 2 magnification. Scale bar, 10μm. [file Image_1.TIF]

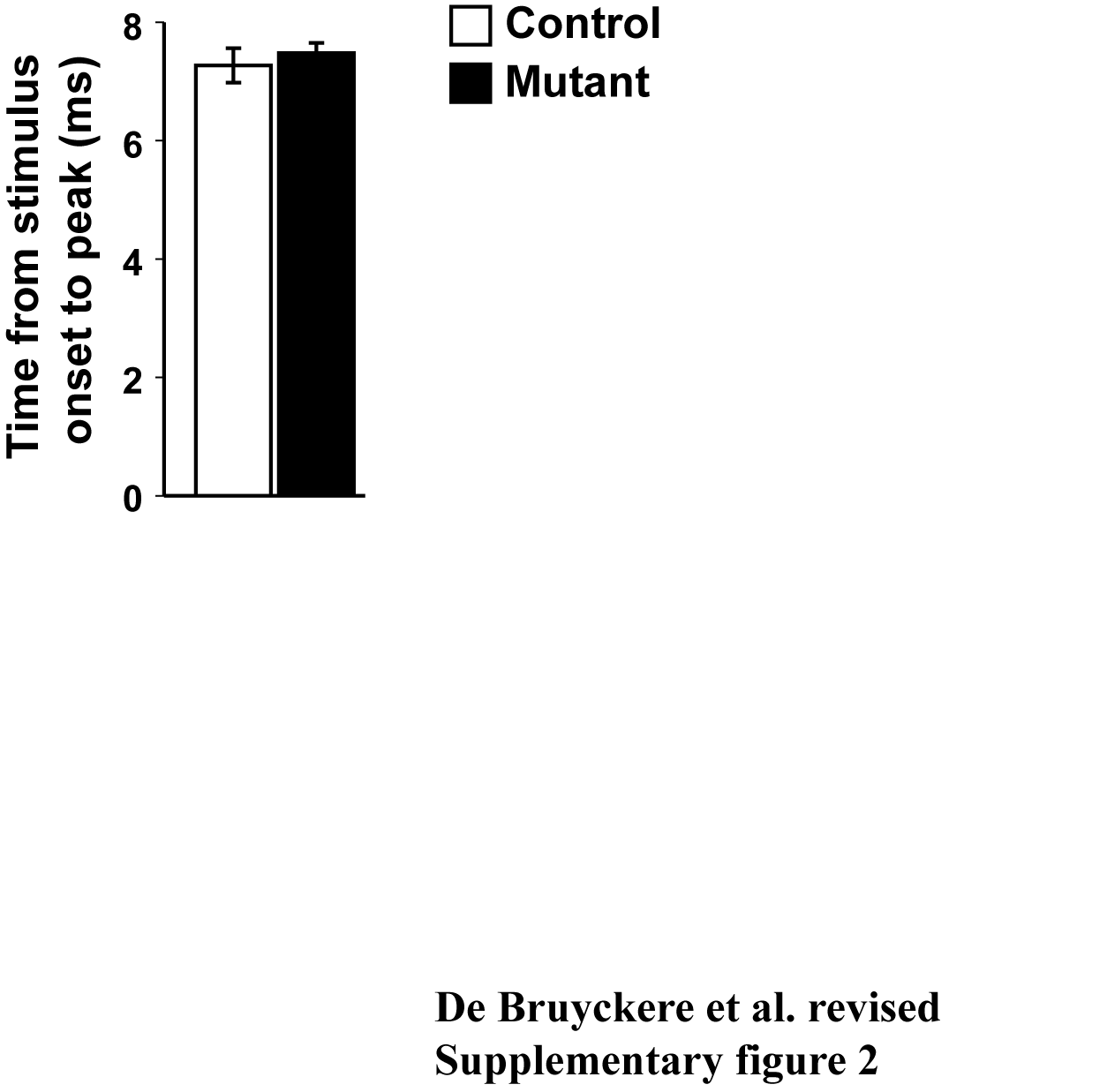

Supplement: FIGURE S2 — Time related analysis of mossy fiber neurotransmission. Measurement of the time separating stimulation of the mossy fiber to fEPSP maximal amplitude. [file Image_2.TIF]
